# Dictionary learning for unsupervised identification of ischemic territories in CP-BOLD Cardiac MRI at rest

**DOI:** 10.1186/1532-429X-17-S1-Q13

**Published:** 2015-02-03

**Authors:** Marco Bevilacqua, Cristian Rusu, Rohan Dharmakumar, Sotirios Tsaftaris

**Affiliations:** 1 IMT Lucca, Lucca, Italy; 2 Northwestern University, Evanston, IL, USA; 3 Cedars-Sinai Medical Center, Los Angeles, CA, USA; 4 The University of Vigo, Vigo, Spain

## Background

Cardiac phase-resolved Blood-Oxygen-Level-Dependent (CP-BOLD) MRI can detect myocardial ischemia at rest without contrast and stress agents. At rest, BOLD myocardial signal intensity varies with cardiac phase: in healthy conditions it is maximal in systole and minimal in diastole, but in disease this pattern is not evident. These changes are not readily visible and post-processing is necessary. Previous methods used segmental analysis and only two images to identify ischemic segments. In this study we demonstrate that it is possible to use unsupervised learning methods to identify ischemia with higher accuracy.

## Methods

Flow and motion compensated 2D short-axis CP-BOLD were acquired at 1.5T along the mid ventricle in 10 canines at baseline and under severe LAD stenosis. Scan parameters were: resolution=1.2x1.2x8mm3, flip-angle=70° and TR/TE =6.2/3.1ms. For each stack, end-systolic (ES) and end-diastolic (ED) images were identified, myocardial borders were traced (in all phases) and segmented in 24 radially consecutive segments, obtaining for each segment a 1D CP-resolved time series. Then time series were arranged column-wise in a matrix Y. The approach relied on the fact that remote to ischemia a cyclic pattern exists (which is found automatically), while in ischemic territories this pattern is not the same. The divergence from this pattern is used to calculate an ischemia likelihood value (ILV). In more details, given Y, a dictionary D and a sparse representation matrix X, such that Y≈DX, are found, the dictionary D consisting of a circulant part accounting for cyclic shifts of the common pattern and a generic one. The approach iterates by finding first D, then X, and then a binary ischemia vector L, by means of the 1-class Support-Vector-Machine algorithm, which establishes if a time series is ischemic (L=1) or not (L=0) by also looking at how good it is represented by the dictionary. At each iteration time series with L=1 are excluded when updating D; the process is repeated till convergence. Finally, ILV, between 0 and 1, is calculated as a function of the support vectors and is used for visualization. Due to lack of gold standard validation of ischemia at rest, accuracy of the proposed and the Systolic to Diastolic Ratio (SDR) approach were compared using synthetic data obtained by a state-of-the-art method.

## Results

**Figure 1:**
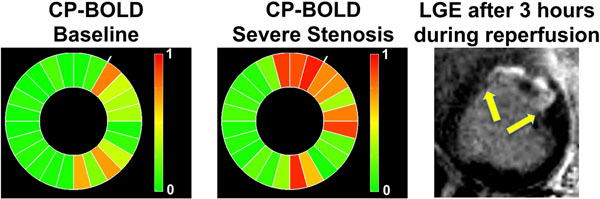
Bulls eye plots visualizing (green no ischemia present, red ischemia) the ischemia likelihood value (ILV) obtained with the proposed approach on a canine with LAD stenosis. Left: baseline (no occlusion), middle: severe stenosis within 20 mins of artery occlusion, right: LGE imaging obtained after 3 hours of occlusion and during reperfusion. An infarct in the LAD territory is seen in LGE. The proposed method finds under ischemia (prior to infarction) within the LAD territory significant deviations (color-coded with red hues) from the remote pattern (ILV values close to 1), which are not present before stenosis (baseline).

In an example from a canine the proposed approach identifies correctly the ischemic territory (Fig. 1). As seen in Table 1, experiments with synthetic data show that the proposed approach obtains superior accuracy when compared to the SDR approach from the literature.

**Table 1:** Synthetic experiments to evaluate the accuracy of the proposed approach in comparison to the SDR approach, which uses only two time points in the time series.

	Number of synthetic time series 100	Number of synthetic time series 150
Proposed approach	92±4%	94±5%

Systolic to Diastolic Ratio (SDR)	72±8%	74±7%

## Conclusions

Although further experiments are necessary to validate this approach, when combined with appropriate visualization and quantification methods, CP-BOLD can open the road to repeatable, truly non-invasive diagnosis of ischemic heart disease, primarily in detecting and triaging patients experiencing acute coronary syndromes and particularly in cases of NSTEMI.

## Funding

US National Institutes of Health, National Heart Lung and Blood Institute, (grant no: 2R01HL091989-05)

Table 1. Synthetic experiments to evaluate the accuracy of the proposed approach in comparison to the SDR approach, which uses only two time points in the time series.

The ratio of ischemic to total time series is known by design and fixed at 33% (i.e., ground truth assignment was known). Accuracy reflects how many time series were assigned a correct status (ischemia or remote) comparing the result of each approach with the ground truth assignment. The number of time series (100 or 150) was varied and several synthetic datasets (200) were generated. The average ± standard deviation over 200 experiments were reported above. Locations of end-systole and end-diastole were fixed. SDR was defined as the ratio of average segmental intensity obtained from the images at systole and diastole. A segment is ischemic if SDR <1. Statistical test (two tailed paired t-test) shows significant difference in performance among the two approaches.

